# Recombinase Polymerase Amplification/Cas12a-Based Identification of *Xanthomonas arboricola* pv. *pruni* on Peach

**DOI:** 10.3389/fpls.2021.740177

**Published:** 2021-11-23

**Authors:** Mei Luo, Fan-Zhu Meng, Qin Tan, Wei-Xiao Yin, Chao-Xi Luo

**Affiliations:** ^1^Key Laboratory of Horticultural Plant Biology, Ministry of Education, Huazhong Agricultural University, Wuhan, China; ^2^Hubei Key Laboratory of Plant Pathology, Huazhong Agricultural University, Wuhan, China

**Keywords:** CRISPR/Cas12a, RPA, *Xanthomonas arboricola* pv. *pruni*, peach, bacterial spot

## Abstract

Peach bacterial spot caused by *Xanthomonas arboricola* pv. *pruni* (*Xap*) is a devastating disease worldwide and frequently causes massive economic losses. In recent years, it has become a pandemic outbreak in most peach production areas of China, especially on precocious peaches in the middle reach of the Yangtze River. Rapid, user-friendly detection is extremely important to make the correct diagnosis and develop suitable control strategies. In this study, we described a recombinase polymerase amplification (RPA)/Cas12a-based system that combines RPA and CRISPR/Cas12a for *Xap* identification. A total of three crRNAs were designed to target a highly conserved ABC transporter ATP-binding protein-encoding gene *ftsX* to make specific detection of *Xap*. Results showed that crRNA 2 and crRNA 3 could get consistent detection for *Xap*. To realize the visualization of detection results, we additionally introduced FQ-reporter and FB-reporter. The developed method was highly sensitive and could detect as low as 10^–18^ M *Xap* gDNA with a mini-UV torch, corresponding to 1.63 copies/μl or 8.855 fg/μl gDNA of *Xap*, while with lateral flow strips, the sensitivity was 10^–17^ M. In addition, this method could specifically detect *Xap* from other closely related bacteria or pathogens associated with peach diseases. Furthermore, this method could make correct identification for *Xap* with crude DNA using NaOH-based extraction (3 min) directly from diseased peach samples. Considering that the developed method could get results within 2 h and could be performed at 37°C (body temperature), it is promising to be applied for *Xap* diagnosis and monitoring in fields.

## Introduction

Bacterial spot caused by *Xanthomonas arboricola* pv. *pruni* (*Xap*) is an economically important disease of peach worldwide and results in huge economic losses on most of the stone fruit trees (Stefani, 2010; Janse, 2012). In recent years, it has become a widespread epidemic issue that broke out into disasters in some orchards, especially on precocious peaches in the middle reach of the Yangtze River where the disease incidence can reach 100% on susceptible cultivars. *Xap* infects not only leaves but also fruits and branches on a wide range of *Prunus* species including peach (*Prunus persica*), plum (*Prunus salicina*), apricot (*Prunus armeniaca*), cherry (*Prunus avium*), and almond (*Prunus amygdalus*), as well as ornamental plants such as *Prunus davidiana* and *Prunus laurocerasus* (Rosello et al., 2012; Tjou-Tam-Sin et al., 2014). The initial symptom on leaves is the appearance of water-soaked spots, then the spots gradually enlarge and become necrotic in the center, eventually falling off to form irregular perforations, and even leading to leaf fall. On fruits, diseased tissue presents deep and wide-shaped cracking, which seriously affects the quality and taste of peaches. These characteristics share similar clinical signs with the peach scab, black spot, and early canker (Bock et al., 2011; Oura et al., 2013), making its diagnosis difficult. Besides, a low isolation rate and similar colony characteristics to other epiphytic bacteria are also obstacles to its diagnosis. Consequently, diagnostic errors occur frequently.

The current recommended international standard for *Xap* diagnosis according to the European and Mediterranean Plant Protection Organization (EPPO) is based on pathogen isolation (European and Mediterranean Plant Protection Organization [EPPO], 2006), which is accurate, but laborious and time-consuming. With the development of molecular technology, new molecular detection tools have been invented for nucleic acid detection, such as PCR, bio-PCR (Park et al., 2010), duplex PCR (Pothier et al., 2011a), multiplex PCR (Pothier et al., 2011b), and real-time PCR (Palacio-Bielsa et al., 2011). Nevertheless, such methods have limitations in realizing visual-readout way, thus are not suitable for local diagnosis (Bai et al., 2019). Recently, isothermal amplification technology started to be popular and made a new focus in nucleic acid detection due to its simplicity and accuracy, such as loop-mediated isothermal amplification (LAMP) and recombinase polymerase amplification (RPA). However, LAMP requires a relatively higher reaction temperature (around 60°C) and is prone to high false positives, which affects its field use (Suleman et al., 2016). RPA is the latest developed isothermal amplification detection technology in the world and is claimed to eventually replace PCR. RPA targets the double-stranded DNA (dsDNA) by recombinase-primer complex and amplifies the target region through strand-displacement DNA synthesis (Piepenburg et al., 2006). It can successfully amplify targeted DNA sequences at 37–42°C for 30 min with high sensitivity. The results can be visualized by combining them with fluorescence signals or lateral flow assay (LFA).

Recently, another new nucleic acid detection tool emerged and switched to a new wave of scientific research. It is the RNA-guided CRISPR/Cas-based nucleic acid detection system that has shown great promise for highly sensitive and rapid nucleic acid detection (Sashital, 2018; Li et al., 2019a). In this system, three nucleases, namely, Cas12a, Cas13a, and Cas14, were generally used (Gootenberg et al., 2017, 2018; Chen et al., 2018; Harrington et al., 2018; Li et al., 2018; Teng et al., 2019). The specially designed crRNA complementary to the target can activate the *cis*- and *trans*-cleavage activities of Cas nuclease, which not only cleave dsDNA target in a specific way but also cleave the non-specific single-stranded DNA (ssDNA) in the whole environment. The CRISPR/Cas system is also evolving into many innovative applications including nucleic acid detection, such as DETECTR, SHERLOCK, and HOLMES (Gootenberg et al., 2017, 2018; Knott and Doudna, 2018; Myhrvold et al., 2018; Li et al., 2019a). They are generally combined with RPA, LAMP, or PCR to amplify the targeting signal. The Cas12a-based DETECTR and HOLMES target dsDNA and detect the signal with fluorescence detection equipment, thus limiting the application under field conditions (Li et al., 2018). Cas13a-based SHERLOCK, both the target and probe are RNAs, which requires an additional *in vitro* transcription step to convert dsDNA to RNA (Gootenberg et al., 2017, 2018). Cas14 targets ssDNA, which also needs extra conversion from dsDNA to ssDNA (Harrington et al., 2018). Overall, Cas12a-based detection has more potential to be used under field conditions with some modifications. Actually, Bai et al. (2019) developed the CORDS (Cas12a-based on-site and rapid detection system), which combined Cas12a with recombinase aided amplification (RAA) and an immunochromatographic lateral flow strip for ASFV on-site detection. Zhang et al. (2020) also used Cas12a combined with RPA and lateral flow strip to detect rice blast pathogen.

In this study, we applied the Cas12a-based system combined with RPA and an ssDNA probe to detect *Xap*, the causal agent of peach bacterial spot. Two visualization approaches were tailored, one is the fluorescence visualization readout and the other is to use lateral flow strips. We found a mini-UV torch, which can detect the fluorescence signal easily instead of the complicated fluorescence detection instrument. The integrated RPA/Cas12a detection procedure was conducted for *Xap* detection from diseased and healthy samples. It could make an accurate detection even using the crude DNA with NaOH-based DNA quick extraction, and the whole detection only took approximately 1 h. As an easy, effective, and field-deployable application, it is anticipatory that this innovative nucleic acid detection method using RPA/Cas12a will be a useful tool for early detection of *Xap* in practice.

## Materials and Methods

### Isolates and Plant Materials

*Xanthomonas arboricola* pv. *pruni* isolate ZY2-3-1a was routinely used in *Xap* detection assays. Two closely related *Xanthomonas* species/subspecies, namely, *Xanthomonas campestris* pv. *campestris* and *X. arboricola* pv. *juglandis*, other nine bacterial species which were isolated and identified from peach samples, and four common fungal species, namely, *Alternaria alternata*, *Venturia carpophila*, *Monilinia fructicola*, and *Wilsonomyces carpophilus* from peach were used in the assessment of the specificity of RPA/Cas12a assay (Supplementary Table 1). All the isolates were identified to the species level based on their morphological characteristics and molecular identification (comparison of ITS sequences for fungal species and 16S rDNA for bacterial species). For RPA/Cas12a assay in field detection, the peach leaf samples were collected from the cultivar Xiahui 5 at an orchard in Huazhong Agricultural University, Wuhan, Hubei Province, China.

### DNA Preparation

For regular genomic DNA preparation, the Rapid Bacterial Genomic DNA Isolation Kit (ToloBio, Shanghai, China) was used to purify DNA according to the instructions of the manufacturer.

For field rapid detection, the NaOH-based crude DNA extraction was used, and 2–4 pieces of diseased or healthy tissues (around 0.6 cm × 2 cm, 50 mg) were ground in a mortar and submerged in 500 μl of 0.5 M NaOH (Qian et al., 2018). After it was blended for 1 min and kept at room temperature for 1–2 min, the supernatant was diluted 50-fold with Tris-EDTA (TE) buffer (10 mM Tris, 1 mM EDTA, pH 8.0) and directly used as a template for the RPA/Cas12a assay.

### Cas12a crRNA Design and Synthesis

CrRNA guide sequence was designed according to the adjacent motif of PAM (TTTV) for the *ftsX* gene which has been described as a *Xap* specific gene in the previous study (Pothier et al., 2011a). In brief, the crRNAs were designed based on the targeting sequence (a 323 bp fragment of *ftsX* gene) at the website https://portals.broadinstitute.org/gppx/crispick/public by simply selecting corresponding PAM sites and Cas12a and then, select candidates based on the resulted scores. For the synthesis of the crRNAs, a DNA oligo (100 μM) (T7-crRNA-oligo) containing T7 promoter, guide sequence, and conserved stem-loop sequence was synthesized at Tianyi Huiyuan, Wuhan Biotechnology Co., Ltd., Wuhan, China. Then, T7-crRNA-oligo and T7-top oligo were annealed [annealing Buffer for DNA Oligos(5X), Beyotime] to generate a partially dsDNA template, which was finally used for crRNA preparation using the HiScribe T7 Quick High Yield RNA Synthesis Kit (New England Biolabs, MA, United States). The crRNA purification was performed using the RNA Clean and Concentrator Kit (New England Biolabs). All crRNAs were examined using a NanoDrop spectrophotometer (Thermo Fisher, Shanghai, China). The DNA oligos for crRNA synthesis are shown in Supplementary Table 2.

### *In vitro* Double-Stranded DNA Substrate Cleavage Assay

To verify the targeted cleavage efficiency of Cas12a combined with different crRNAs, *in vitro* dsDNA substrate cleavage assay was carried out using the *ftsX* gene PCR product, LbCas12a (New England Labs), and purified crRNAs. The total volume is 10 μl containing 250 nM LbCas12a, 500 nM crRNA, 1×NEBuffer 2.1 (New England Biolabs), and 5 μl PCR product. The reaction was performed at 37°C for 1 h, and the cleavage products were analyzed by 1% agarose gel electrophoresis.

The *ftsX* gene PCR amplification was performed in a 25 μl reaction mixture using primer pair XapY17-F and XapY17-R (Tianyi Huiyuan) for 1 μl of each primer (10 μM) (Supplementary Table 3), 1 μl of total DNA (10 ng/μl), 12.5 μl of 2 × *Taq* PCR MasterMix (YEASEN, Wuhan, China), and 9.5 μl of double-distilled water, which was carried out at 94°C for 4 min followed by 35 cycles of 94°C for 30 s, 55°C for 30 s, 72°C for 60 s, and a final extension of 72°C for 10 min. The 5 μl PCR products were separated on 1% agarose gels containing gel-red (US Everbright Inc., Suzhou, China) in 1 × TAE buffer and visualized under UV light.

### Recombinase Polymerase Amplification Assay

The species-specific *ftsX* gene was selected to detect *Xap*. Three pairs of *ftsX*-specific primers were designed for RPA assay with Primer3^1^ according to RPA primer design rules. The primer sequences are available in Supplementary Table 3.

The RPA assay was processed using the TwistAmp Basic Kit (TwistDx, Beijing, China) according to the manufacturer instructions in 50 μl volumes containing 29.5 μl of rehydration buffer, 12.2 μl of nuclease-free H_2_O, 480 nM of forward and reverse primers (Tianyi Huiyuan), and 1 μl of genomic DNA (10 ng/μl), which was mixed with freeze-dried RPA enzyme powder. Finally, 2.5 μl of magnesium acetate (280 nM) was added to activate the reaction and incubated at 37°C for 30 min. The RPA products were purified using the DNA Purification Kit (Tianmo Biotech, Beijing, China) and analyzed by 1% agarose gel electrophoresis.

### Establishment of the Recombinase Polymerase Amplification/Cas12a-Fluorescence Assay

Generally, an ssDNA probe is needed for the establishment of the RPA/Cas12a analysis to show the presence of the target signal. An FQ-reporter (Tianyi Huiyuan) (Supplementary Table 4) with a FAM fluorophore and a quencher was added to establish the RPA/Cas12a-fluorescence assay, which contained 250 nM LbCas12a, 500 nM crRNA, 1 × NEBuffer 2.1, 500 nM FQ-reporter, and 2–5 μl RPA reaction products in total 10 μl volumes. Reactions were performed at 37°C for 1 h, and the fluorescence intensity was measured on a fluorescence plate reader in the FAM channel. One-way ANOVA with Dunnett’s post-test set at *p* < 0.05 was used to assess positive results.

For optimizing the concentration ratio of LbCas12a to crRNA, reactions were performed as described above with the following modifications: the LbCas12a concentration was set at 50 nM, the crRNA concentrations were set at 25, 50, 100, 200, and 300 nM for ratio gradients. For optimizing the concentration of LbCas12a, four concentration gradients of 50, 150, 250, and 350 nM were set according to the optimal ratio of LbCas12a to crRNA. The FQ-reporter concentrations were set to 500, 1,000, 1,500, and 2,000 nM for optimal visualization concentration. Furthermore, a mini-UV torch was used to directly visualize the fluorescence signal with the naked eyes.

Finally, the optimized RPA/Cas12a-fluorescence assay was applied to test the sensitivity using a series of concentration gradients from 1 × 10^–11^ to 1 × 10^–19^ M of the dsDNA targets with three replicates. In addition, two closely related *Xanthomonas* species/subspecies, nine bacterial species which were collected and identified from peach samples, and four common peach fungal species were examined for assessment of its specificity (Supplementary Table 1).

### Establishment of the Recombinase Polymerase Amplification/Cas12a-Lateral Flow Assay

The lateral flow strip was introduced into RPA/Cas12a analysis to resolve restrictions under some situations and improve the applicability. This assay can display the positive band signal at a specified location by binding antibodies and antigens (Supplementary Table 4).

The RPA/Cas12a-LFA assay was carried out according to the optimized conditions of RPA/Cas12a-fluorescence assay. The difference in FQ-reporter was replaced by FB-reporter (Tianyi Huiyuan), the concentration remained 1,000 nM, and other components were unchanged. RPA/Cas12a-LFA reactions were performed at 37°C for 1 h. Finally, 100 μl HybriDetect assay buffer was added and incubated at room temperature for 3 min. Then, the lateral flow strip (TwistDx) was immersed in the reaction tube, and the visible color can be developed in the test band and control band within 2–3 min. The sensitivity and specificity of the RPA/Cas12a-LFA assay were the same as the RPA/Cas12a-fluorescence assays.

### Evaluation of the Recombinase Polymerase Amplification/Cas12a Assay With Inoculated and Naturally Infected Samples

Healthy peach leaves were artificially scratched (0.5 cm) and inoculated with 10^8^ CFU/ml *Xap* bacterial suspension, which was derived from a 2-day-old culture grown in NA liquid culture medium at 28°C. The scratched peach leaves were kept at 28°C for 5–7 days to monitor disease development. The sterile culture solution was used to inoculate the scratched peach leaves as the negative control. A total of five *Xap* strains were inoculated, and the test was repeated three times. Finally, peach leaf tissues were collected for the NaOH-based DNA crude extraction (3 min), which was directly used for the RPA/Cas12a assay.

Similarly, the naturally infected samples were collected from a peach orchard in Huazhong Agricultural University in Wuhan, Hubei Province, China. They were first identified by *ftsX* gene PCR and then tested with the RPA/Cas12a assay as described above.

## Results

### LbCas12a Exhibited Targeted Double-Stranded DNA and Non-targeted Single-Stranded DNA Cleavage Activity

**FIGURE 1:**
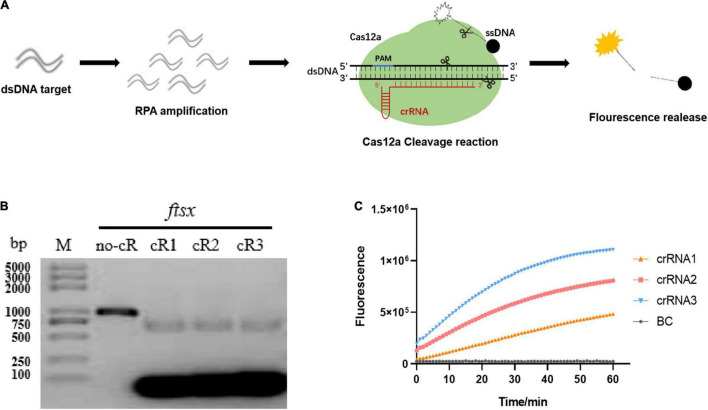
Cas12a-based cleavage reaction combined with recombinase polymerase amplification (RPA) and an FQ-reporter. **(A)** The schematics of Cas12a-based DNA detection using a FQ-reporter. The starburst indicates fluorophore (e.g., FAM), and the black cycle indicates the quencher of the FQ-reporter. **(B)** *In vitro* dsDNA substrate cleavage assay with different crRNAs. M, DNA marker; no-cR, control reaction without crRNA. **(C)** Detection of the RPA/Cas12a-FQ-reporter assay with different crRNAs. BC, blank control with H_2_O.

In the beginning, the optimization of RPA parameters including specific primer screening, incubation time, and reaction temperature were performed. It was observed that *Xap* could be specifically detected by RPA1F/RPA1R primers with high detection efficiency, but other primer sets had all attained weak bands for negative controls. The optimization of reaction conditions only needs to incubate the reactants at 37°C for 15 min (the saturation value) to achieve a fairly high amplification efficiency (Supplementary Figure 1). Then, a total of three crRNAs were designed and synthesized based on the RPA1F/RPA1R targeting sequence for Cas12a cleavage reaction. The schematics of Cas12a-based cleavage reaction using an FQ-reporter were shown in Figure 1A, and the fluorescence signal generated by FQ-reporter degradation indicates the presence of target nucleic acid. The *in vitro* cleavage reaction illustrated that the crRNA 1, crRNA 2, and crRNA 3 guided LbCas12a had a high cleavage activity for targeted dsDNA, almost attained 100% (Figure 1B). The RPA/Cas12a-based DNA test using an FQ-reporter displayed that the non-targeted ssDNA could also be cleaved by LbCas12a guided by different crRNAs, with the efficacy of crRNA 3 > crRNA 2 > crRNA 1 (Figure 1C). However, in several repeated experiments, we found that the non-targeted ssDNA cleavage activity of LbCas12a guided by crRNA3 was unstable. Therefore, the stable crRNA 2 guided Cas12a-based DNA detection was applied in subsequent experiments.

### Establishment of the Recombinase Polymerase Amplification/Cas12a-Fluorescence Assay

**FIGURE 2:**
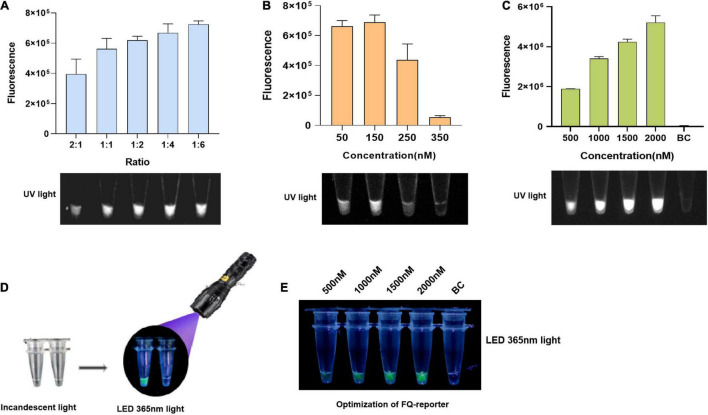
Establishment of the RPA/Cas12a-fluorescence assay. **(A)** End point of Cas12a-fluorescence assay at different LbCas12a/crRNA ratios. **(B)** Optimization of LbCas12a concentration. **(C)** Optimization of FQ-reporter concentration. **(D)** Visualization with a mini-UV torch. **(E)** Optimized visualization results of FQ-reporter with a mini-UV torch. The UV light images were taken by the gel imaging analysis system.

First, we set Cas12a at a lower concentration of 50 nM for more obvious distinguishments of the LbCas12a/crRNA ratios optimization. Optimized results indicated that the ratio of LbCas12a to crRNA was negatively correlated with the accumulation of fluorescence signal, and the smaller the ratio, the higher the accumulation of fluorescence signal (Figure 2). Considering that accuracy and reagent cost, the ratio of LbCas12a to crRNA was used as 1:2 (Figure 2A). For the optimization of Cas12a concentration, the fluorescence signal intensity showed a trend of increasing first and then decreasing from 50 to 350 nM, reaching a peak at 150 nM (Figure 2B). Thus, the concentration of LbCas12a was used at 150 nM for the fluorescence assays in subsequent experiments. Furthermore, we also tried to achieve the naked-eye observation of fluorescence signals with a mini-UV torch (Figure 2D). Through optimizing FQ-reporter concentration, it was shown that the green fluorescence signal could be clearly observed when the concentration of FQ-reporter exceeds 1,500 nM, and the best result was observed at 2,000 nM using both a fluorescence plate reader and a mini-UV torch (Figures 2C,E). Thus, the concentration of FQ-reporter was used at 2,000 nM for the fluorescence assay. Finally, the optimized Cas12a system was combined with the optimized RPA to establish the RPA/Cas12a-fluorescence assay.

### Sensitivity and Specificity of the Recombinase Polymerase Amplification/Cas12a-Fluorescence Assay

**FIGURE 3:**
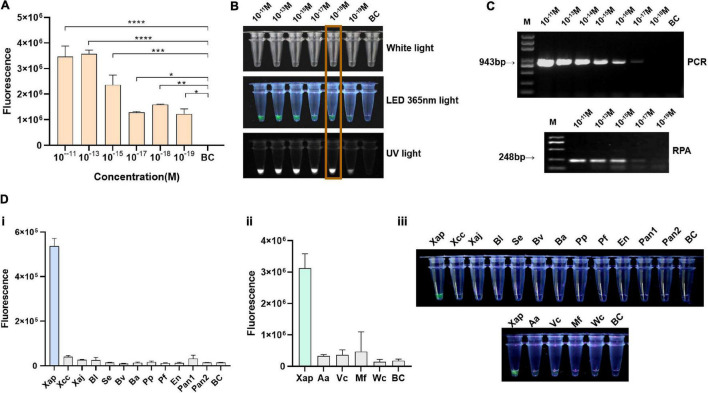
Sensitivity and specificity of the RPA/Cas12a-fluorescence assay. **(A)** Sensitivity of the RPA/Cas12a-fluorescence assay. **(B)** Visualized results of the RPA/Cas12a-fluorescence assay. **(C)** Sensitivity of the PCR and RPA assay. BC, blank control. **(D)** (i) Specificity assessment of RPA/Cas12a-fluorescence assay with *Xap* and bacterial species; (ii) specificity assessment of RPA/Cas12a-fluorescence assay with *Xap* and other fungal species from the peach; and (iii) visualization of (i) and (ii) with a torch at 365 nm light.

The ZY2-3-1a gDNA was diluted from 1 × 10^–11^ M to 1 × 10^–19^ M for sensitivity evaluation. Results showed that the limitation of the RPA/Cas12a-fluorescence assay was 10^–18^ M, corresponding to 1.63 copies/μl or 8.855 fg/μl gDNA of *Xap* (Figures 3A,B), while PCR and RPA assay using the same gDNA had the same detection limitation of 10^–17^ M of input, equivalent to 16.3 copies/μl or 88.55 fg/μl (Figure 3C). Therefore, the RPA/Cas12a assay showed better performance than PCR or RPA assay.

For specificity evaluation, the extracted genomic DNAs of two related *Xanthomonas* species and subspecies, eight bacterial species from peach leaves, and four common fungal species from peach were tested using the RPA/Cas12a-fluorescence assay. Results showed that *Xap* could be specifically detected with relatively high fluorescence signal and visualized by the naked eyes, whereas no or low background signal closed to the blank control for the other tested control strains (Figure 3D).

### Establishment of the Recombinase Polymerase Amplification/Cas12a-Lateral Flow Assay

**FIGURE 4:**
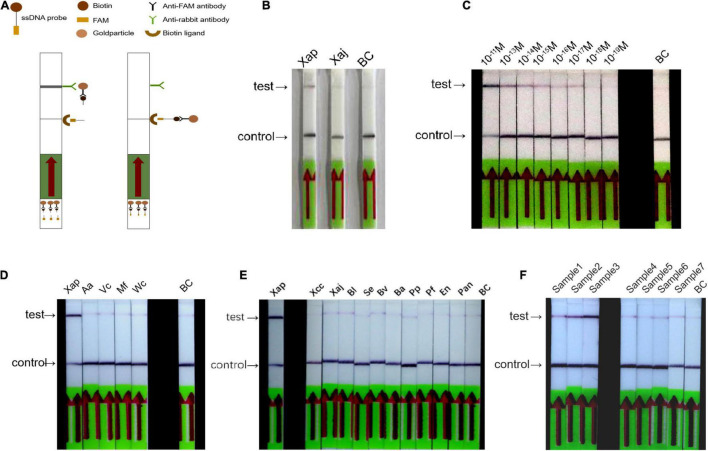
The RPA/Cas12a-LFA assay. **(A)** Schematics of RPA/Cas12a-LFA assay. **(B)** The preliminary establishment of the RPA/Cas12a-LFA system. **(C)** Sensitivity of the RPA/Cas12a-LFA assay. **(D,E)** Specificity of the RPA/Cas12a-LFA assay. **(F)** Field detection of the RPA/Cas12a-LFA assay. BC, blank control.

The RPA/Cas12a-LFA assay was conducted as shown in Figure 4A, the FQ-reporter was substituted with the FB-reporter, and other reagents remained the same as the RPA/Cas12a-fluorescence assay. The RPA/Cas12a-LFA assay was initially established and tested with *Xap*, *Xaj*, and ddH_2_O and produced the expected black band in the test line only for *Xap* (Figure 4B). Similarly, the sensitivity and specificity of the RPA/Cas12a-LFA assay were evaluated in the same way as the RPA/Cas12a-fluorescence assay. The sensitivity of the RPA/Cas12a-LFA assay was tested with 10-fold dilution series (from 1 × 10^–11^ M to 1 × 10^–19^ M) gDNA. Results showed that the sensitivity reached 10^–17^ M, which was 10 times lower than that of the RPA/Cas12a-fluorescence assay (Figure 4C). However, similar to the RPA/Cas12a-fluorescence assay, the RPA/Cas12a-LFA assay also could specifically detect *Xap* from the other 14 control strains (Figures 4D,E).

### Field Evaluation of the Simplified Recombinase Polymerase Amplification/Cas12a Assays

Generally, disease diagnosis mainly happens in fields, where complicated equipment cannot be obtained. Therefore, it is crucial to simplify experimental procedures and develop methods, which do not need complicated and expensive equipment. In view of these requirements, we tried to simplify the detection procedure which could be used for simple diagnosis from disease samples directly without DNA purification.

**FIGURE 5:**
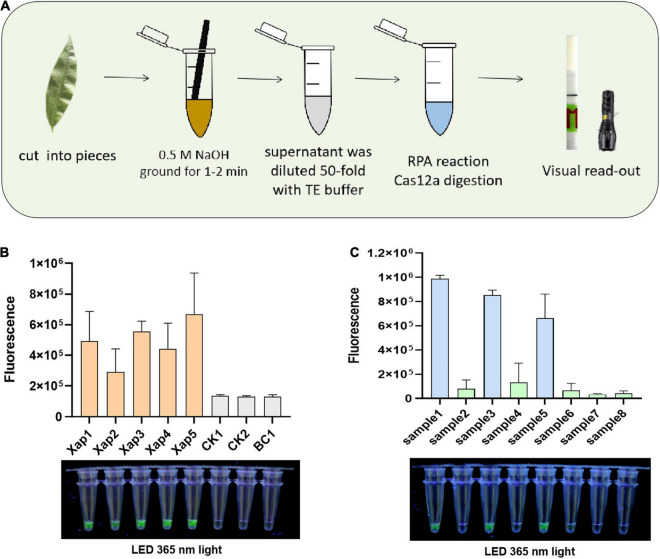
Field evaluation of the RPA/Cas12a-fluorescence assay. **(A)** Schematic of the integrated RPA/Cas12a analysis. **(B)** The RPA/Cas12a-fluorescence detection of inoculated leaves. **(C)** The RPA/Cas12a-fluorescence detection of natural leaves. BC, blank control.

The crude DNA from the NaOH-based extraction methodology was applied to form a simplified RPA/Cas12a detection (Figure 5A). Artificially inoculated and naturally infected peach leaves were directly tested with the simplified RPA/Cas12a detection. Results showed that diseased leaves inoculated with five *Xap* strains all showed positive results, while peach leaves inoculated with the culture medium did not show any symptoms, and there was no obvious accumulation of fluorescence in the simplified RPA/Cas12a assays (Figure 5B). Similarly, the analogous fluorescence was only observed in the infected samples when natural peach leaves were investigated (Figure 5C).

**FIGURE 6:**
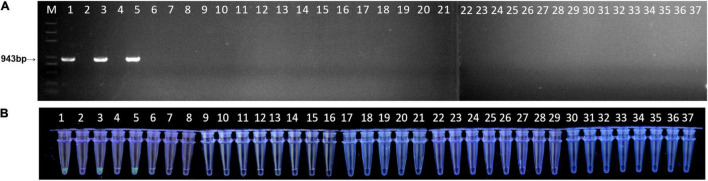
Field detection of natural peach leaves with the simplified RPA/Cas12a-fluorescence assay. **(A)** A total of 37 peach leaf samples were investigated with PCR amplification. **(B)** The simplified RPA/Cas12a-fluorescence assay was applied for the same 37 leaf samples.

To further test the feasibility of the simplified RPA/Cas12a assay, the 37 natural peach leaf samples were collected and tested from an orchard in Huazhong Agricultural University. As expected, the RPA/Cas12a-fluorescence assay exhibited consistent results with *ftsX* gene PCR, 3 positive samples, and 34 negative samples (Figure 6). Then, three positive samples and five randomly selected negative samples were picked for simultaneous verification using the simplified RPA/Cas12a-LFA assay, and consistent results were observed (Figure 4F). Consequently, the simplified RPA/Cas12a assay was practicable for *Xap* diagnosis and monitoring of bacterial spot disease.

## Discussion

In recent years, the peach bacterial spot has caused huge economic losses in China. This disease occurred as a rapid outbreak across the nation, especially in the middle reach of the Yangtze River where there is long-term rainfall and high humidity. Considering its severity and explosiveness, accurate and fast diagnosis of the peach bacterial spot is important and urgent for surveillance and control of its outbreaks. However, current diagnostic technology often requires laboratory facilities and delivery of samples to a qualified laboratory, which delays the progress of diagnosis and increases the probability of a pandemic. Therefore, it is necessary to innovate a simple, fast, and efficient tool, which can not only realize rapid and fast diagnosis under field conditions but also maintain high accuracy.

Recently, CRISPR/Cas-based diagnostics is emerging as a promising technology for nucleic acid detection owing to its simplicity and sensitivity (Zhang et al., 2020). Obviously, a variety of applications have been derived, such as the diagnosis of African swine fever (Wang et al., 2020), HPV16 and HPV18, the causal agent of cervical cancer (Mukama et al., 2020), new coronavirus disease 2019, etc. (Broughton et al., 2020). In addition, related applications in plants have also been proposed. For instance, Cas12a is engineered to detect the virus of grapevine and CaMV35S promoter in genetically modified soybeans (Li et al., 2019b; Wu et al., 2019). Zhang et al. (2020) first utilized the CRISPR/Cas12a system to realize the fast detection of the rice blast pathogen and GMO in rice. In view of these, we can anticipate that this Cas12a-based DNA detection method could be broadly used in disease diagnosis with some modifications.

The crRNA is critically important for Cas12a-based detection. In this study, a total of three crRNAs were designed for the Cas12a cleavage reaction. Surprisingly, all of them successfully degraded the dsDNA target *in vitro*. We guessed that this may rely on CRISPR/Cas12a (*Cpf1*) which uses a single RuvC catalytic domain for guide RNA-directed dsDNA cleavage. The compatibility of crRNA and dsDNA affects the stability of Cas12a cleavage conformation, which determines products with different lengths (Zhang et al., 2019). LbCas12a exhibited the non-target ssDNA cleavage reaction which was revealed using an FQ-reporter, and all crRNAs appeared obvious fluorescence signal accumulation, while signal intensities vary frequently, such as crRNA3. It is possible because the targeting sequence of crRNA plays a key role to determine the editing efficiency in Cas12a-based genome editing (Kim et al., 2017). Finally, we chose a stable crRNA2 for subsequent innovations. This fully illustrates the necessity of designing multiple crRNAs simultaneously for the Cas12a-based test against specific targets.

Based on a series of experiments, we developed the RPA/Cas12a assays for peach *Xap* detection. We further simplified the RPA/Cas12a analysis with the crude DNA from peach leaves directly, which realized the *Xap* detection at body temperature within 2 h. Actually, this is the first report based on RPA or Cas12a system for *Xap* detection. We provided two visualization approaches with a mini-UV torch or immunochromatographic lateral flow strips so that the operators can adjust the experimental scheme freely according to the available conditions on-site, which greatly enhanced the detection practicability and resolved some bottlenecks that could not be conducted previously under field conditions. This new assay comprises two steps, RPA reaction first recognizes and amplifies target signals, and Cas12a-based dsDNA targets and non-target ssDNA cleavage reaction forms the secondary detection which effectively avoids false-positive errors and ensures high accuracy. Unlike other technologies, the simplified RPA/Cas12a assays do not require complex instruments, only 37°C incubation for 1 h, which can be easily attained by the body temperature under field conditions. Furthermore, the RPA/Cas12a assay maintains high sensitivity of more than 10^–17^ M of *Xap* gDNA and high specificity which could accurately distinguish the *Xap* from other peach pathogens, even from the closely related pathovar *Xaj* in the same species *X. arboricola*. Considering that the whole test procedure is easy, rapid, flexible, and effective, peach growers or other users with basic knowledge can easily carry out the test and analyze the result under field conditions. We have full confidence that this technology will soon reach the greatest degree of universal application in practice. To further enhance the usage of this technology in future, corresponding detection kits should be produced.

Certainly, there are also some potential limitations and future improvements that should be noted. First, in the specificity of the RPA/Cas12a-fluorescence assay or the RPA/Cas12a-LFA assay, the background fluorescence signal is occasionally too high which may cause false-positive results. In this case, reducing the concentration of the FQ-reporter might be a way to solve this issue. Although it is still unclear, some reasonable conjectures could be raised. Mukama et al. (2020) has shown that the length and sequence of FQ-reporter affect the non-specific ssDNA cleavage activity of Cas12a. The RNA-independent nuclease activities and off-targeting effect of Cas12a-based genome editing may be related to this, leading to some false positive situations. In addition, the aerosol pollution caused by RPA amplification may lead to some background signals. Second, RPA targeting sequences should be rich in multiple PAM sites so that multiple crRNAs can be designed for Cas12a cleavage reaction. Finally, based on the observation of the fluorescence curve, it only took 30 min to achieve a fairly high fluorescence accumulation, and the reaction gradually reached a plateau. The field evaluation of the RPA/Cas12a assay illustrated that obvious fluorescence signals could also be observed within 30 min. However, the shorter cleavage time may cause false-negative results in the detection of low biomass DNA samples, and the detection sensitivity may be significantly reduced. Actually, the sensitivity of diagnosis based on the LFA strip can be significantly reduced to 10^–13^M when the reaction time is reduced to 30 min (Supplementary Figure 2). To obtain accurate results, we suggested that the Cas12a cleavage reaction can be carried out for 1 h. In view of this, the NaOH-based DNA crude extraction should be further optimized to increase the DNA yield and purity so that *Xap* can be detected even at the early stage of the low bacterial population. Besides, we can appropriately shorten the reaction time to 30 min to improve detection efficiency wherever good sample quality is available.

## Conclusion

We developed an easy, fast, and effective assay for *Xap* detection. It could achieve rapid detection of *Xap* at 37°C within 2 h, and two flexible approaches have been designed for users to choose. We also launched the simplified RPA/Cas12a assay program, which can realize batch sample detection directly under field conditions. Based on the advantages and advancements, we believed that this RPA/Cas12a assay will soon be introduced into commercial use in practice.

## Data Availability Statement

The original contributions presented in the study are included in the article/Supplementary Material, further inquiries can be directed to the corresponding author.

## Author Contributions

C-XL conceived the idea and finalized the manuscript. C-XL and ML designed the experiments. ML, F-ZM, and QT performed the experiments and analyzed the data. ML, W-XY, and C-XL wrote the manuscript. All authors contributed to the article and approved the submitted version.

## Conflict of Interest

The authors declare that the research was conducted in the absence of any commercial or financial relationships that could be construed as a potential conflict of interest. The handling editor declared a past collaboration with one of the authors W-XY.

## Publisher’s Note

All claims expressed in this article are solely those of the authors and do not necessarily represent those of their affiliated organizations, or those of the publisher, the editors and the reviewers. Any product that may be evaluated in this article, or claim that may be made by its manufacturer, is not guaranteed or endorsed by the publisher.

## Footnotes

1
http://bioinfo.ut.ee/primer3/
