# Supplementary material for: Recombinase Polymerase Amplification/Cas12a-Based Identification of Xanthomonas arboricola pv. pruni on Peach
Source: Front Plant Sci. 2021 Nov 23;12:740177. doi: 10.3389/fpls.2021.740177 (PMC8650578; doi:10.3389/fpls.2021.740177)
Supplement: Supplementary file 1 [file Data_Sheet_1.docx]

Supplementary Material

# **Supplementary Table 1.** Isolates used in this study

| Isolates | Taxonomy | Abbreviation | Location | Year |
| --- | --- | --- | --- | --- |
| ZY2-3-1a | *Xanthomonas arboricola*pv*.pruni* | Xap | Zaoyang,Hubei | 2017 |
| CQ1 | *Xanthomonas campestris*pv*.campestris* | Xcc | Chongqing | 2018 |
| HTY1 | *Xanthomonas arboricola*pv*.junglandis* | Xaj | Enshi,Hubei | 2017 |
| HBWH2 | *Bacillus licheniforms* | Bl | Wuhan,Hubei | 2020 |
| HBWH5 | *Staphylococcus epidermidis* | Se | Wuhan,Hubei | 2020 |
| LZ-7-7 | *Bacillus velezensis* | Bv | Linzhi,Xizang | 2019 |
| LZ-5-10 | *Bacillus amyloliquefaciens* | Ba | Linzhi,Xizang | 2019 |
| HBCL612 | *Pseudomonas putida* | Pp | Changli,Hebei | 2018 |
| HBCL762 | *Pseudomonas fulva* | Pf | Changli,Hebei | 2018 |
| HBCL241 | *Enterobacter*sp*.* | En | Changli,Hebei | 2018 |
| GXXY2-6-1 | *Pantoea agglemerans* | Pan | Guilin,Guangxi | 2017 |
| ZY2-7-1a | *Pantoea agglemerans* | Pan | Zaoyang,Hubei | 2017 |
| LNBLF2-2 | *Alternaria armeniacae* | Aa | Dalian,Liaoning | 2019 |
| HBWH28 | *Venturia carpophila* | Vc | Wuhan,Hubei | 2019 |
| YHC11-8c | *Monilinia fructicola* | Mf | Honghe,Yunnan | 2011 |
| XJHG1 | *Wilsonomyces carpophilus* | Wc | Shihezi,Xinjiang | 2019 |

**Supplementary Table 2.** Cas12a targeting sequence and DNA oligos for *in vitro* transcription of crRNAs

| **Cas12a targeting sequence** | | |
| --- | --- | --- |
| crRNA name | Guide sequence (5’-3’) | PAM (5’-3’) |
| cR1 | UCUCGAAGAUCAUGCUGAGCAAG | TTTA |
| cR2 | AAGCUGCUUGGCAUAACGCCCUU | TTTG |
| cR3 | GGUUUAUCUCGAAGAUCAUGCUG | TTTG |
| **DNA oligos for in vitro transcription of crRNAs using T7 polymerase** | | |
| T7-top | gaaattaatacgactcactataggg | T7 promoter sequence |
| T7-cR1-oligo | cttgctcagcatgatcttcgagaATCTACAACAGTAGAAATT*CCCTATAGTGAGTCGTATTAATTTC* | Oligonucleotide for the *in vitro* transcription of cR1, pairing with T7-top |
| T7-cR2-oligo | aagggcgttatgccaagcagcttATCTACAACAGTAGAAATT*CCCTATAGTGAGTCGTATTAATTTC* | Oligonucleotide for the *in vitro* transcription of cR2, pairing with T7-top |
| T7-cR3-oligo | cagcatgatcttcgagataaacc*ATCTACAACAGTAGAAATTCCCTATAGTGAGTCGTATTAATTTC* | Oligonucleotide for the *in vitro* transcription of cR3, pairing with T7-top |

**Supplementary Table 3.** Primers used in this study

| **Primer name** | **sequence (5’-3’)** |
| --- | --- |
| RPA1F | GTCTCAGCGTCAGTTCTAGCATGTTCTCGTG |
| RPA1R | GATCAAATAATCTTCAAGTCCCTCCAAACGGA |
| RPA2F | CTCGTTTCCACACCGATTTGCAGAAGGATAC |
| RPA2R | CTTGCTGGTCGACATAACTTTGAAGCTGCTTG |
| RPA3F | CAAGCAGCTTCAAAGTTATGTCGACCAGCAAG |
| RPA3R | CAACGCACAGATATTGACCATGCAAACAGTC |
| XapY17-F | GACGTGGTGATCAGCGAGTCATTC |
| XapY17-R | GACGTGGTGATGATGATCTGC |

**Supplementary Table 4.** Sequences of synthesized DNA probes used in this study

| Primer name | sequence (5’-3’) | Purpose |
| --- | --- | --- |
| FQ-reporter | 5’-6-FAM -ttatt-Quencher-3’ | ssDNA reporter for fluorescence detection |
| FB-reporter | 5’-6-FAM -ttattttattttatt-Biotin-3’ | ssDNA reporter for lateral flow assay |


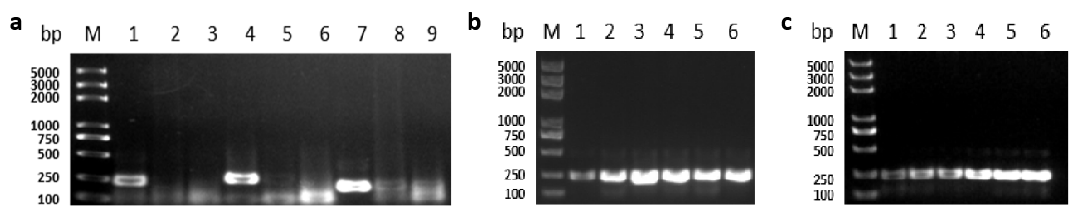


**Supplementary Figure 1.** Establishment of the RPA assay. **a.** Optimization of three pairs of RPA primers, RPA1F/1R: lanes 1, 2, 3, RPA2F/2R: lanes 4, 5, 6, RPA3F/3R: lanes 7, 8, 9; Lanes 1, 4, 7: *Xap*, Lanes 2, 5, 8: *Xaj,*  Lanes 3, 6, 9: *Pantoea agglemerans*. **b**. Optimization of the incubation time: lanes 1, 2, 3, 4, 5 and 6 represent the incubation time of 5, 10, 15, 20, 25, and 30 min. **c**. Optimization of incubation temperature, lanes 1, 2, 3, 4, 5 and 6 represent the incubation temperature of 30, 33, 35, 37, 39, and 42℃.


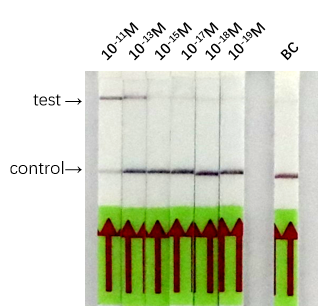


**Supplementary Figure 2.** The sensitivity of RPA-Cas12a-LFA analysis incubation for 30 minutes.
